# Meta-Analysis of Relationships between Human Offtake, Total Mortality and Population Dynamics of Gray Wolves (*Canis lupus*)

**DOI:** 10.1371/journal.pone.0012918

**Published:** 2010-09-29

**Authors:** Scott Creel, Jay J. Rotella

**Affiliations:** Department of Ecology, Montana State University, Bozeman, Montana, United States of America; Northeastern University, United States of America

## Abstract

Following the growth and geographic expansion of wolf (*Canis lupus*) populations reintroduced to Yellowstone National Park and central Idaho in 1995–1996, Rocky Mountain wolves were removed from the endangered species list in May 2009. Idaho and Montana immediately established hunting seasons with quotas equaling 20% of the regional wolf population. Combining hunting with predator control, 37.1% of Montana and Idaho wolves were killed in the year of delisting. Hunting and predator control are well-established methods to broaden societal acceptance of large carnivores, but it is unprecedented for a species to move so rapidly from protection under the Endangered Species Act to heavy direct harvest, and it is important to use all available data to assess the likely consequences of these changes in policy. For wolves, it is widely argued that human offtake has little effect on total mortality rates, so that a harvest of 28–50% per year can be sustained. Using previously published data from 21 North American wolf populations, we related total annual mortality and population growth to annual human offtake. Contrary to current conventional wisdom, there was a strong association between human offtake and total mortality rates across North American wolf populations. Human offtake was associated with a strongly additive or super-additive increase in total mortality. Population growth declined as human offtake increased, even at low rates of offtake. Finally, wolf populations declined with harvests substantially lower than the thresholds identified in current state and federal policies. These results should help to inform management of Rocky Mountain wolves.

## Introduction

### Status of US Wolf Populations

Following their extirpation by direct harvesting across most of the United States, gray wolves (*Canis lupus*) were among the 14 mammals originally listed by the U.S. Fish and Wildlife Service under the Endangered Species Preservation Act of 1966. This legal protection was renewed under the Endangered Species Act of 1973, and wolves are now considered endangered in 16 states. Following steady growth of the wolf population of the Western Great Lakes region, this population segment was down-listed to threatened status in 1978. A proposal for delisting in Minnesota and Michigan was initiated in 2000 and remains under legal appeal. Following reintroduction into Yellowstone National Park and central Idaho in 1995–1996, wolves in the Northern Rocky Mountains Recovery Area grew to a minimum of 1,645 wolves at the end of 2008 [1]. This population segment (including all or parts of Idaho, Montana, Oregon, Utah, Washington, Wyoming) was delisted in 2009 [2], a decision that also remains under appeal. Legal authority for wolf management passed from the US Fish and Wildlife Service to state agencies in this region, and public hunting seasons were initiated in Idaho and Montana, with quotas of 255 (220+35 within the Nez Perce Tribal Treaty Area) and 75 wolves, respectively [3]–[6]. These quotas represent an annual harvest of 20% of the regional population. Quotas were filled in 7 of 12 Idaho regions with a total harvest of 188 wolves. Montana's wolf season closed after 23 days with the quota 96% filled. Together with wolves killed in predator control operations (145 in Montana and 93 in Idaho), humans killed 44% of Montana's wolves and 37.1% of the two-state population in 2009. In March 2010, Montana liberalized its policy for control of wolves that prey on livestock, no longer requiring confirmation by state wildlife officials before wolves near livestock carcasses are trapped or shot. In July 2010, Montana increased the public hunting quota by a factor of 2.5, from 75 to 186 wolves. Idaho is now considering similar changes to wolf management policy.

Predator control and sport hunting are well-established tools to manage large carnivores and broaden societal acceptance of wolves, but to our knowledge it is unprecedented for a species to move this rapidly from highly protected to heavily-hunted, and it remains important to quantitatively assess the probable consequences of these policies as carefully as possible (regardless of the intended outcome). In general, stakeholders calling for reductions in wolf numbers are concerned about three issues: livestock losses, effects on ungulates (particularly elk) and human safety. In 2008 and 2009, Northern Rocky Mountain wolves were responsible for an average of 203 confirmed kills of cattle (from a population of approximately 5.9 million cattle) and 538 confirmed kills of sheep, or 0.8 cows/wolf pack/year and 2.2 sheep/wolf pack/year [1]. Elk numbers in some areas have declined in parallel with wolf recolonization, particularly in locations with locally high wolf density such as portions of the Greater Yellowstone Ecosystem [7], [8], though elk numbers have remained stable or increased in many other areas during the period of wolf recovery [9]. For example, 60% of Montana elk management units were above target population density in 2002, despite liberalized hunting regulations [9]. Wolves have not killed or physically injured people in the Northern Rocky Mountains (NRM) since reintroduction. Current state policies for NRM wolf management focus mainly on providing hunting opportunity, reducing population sizes, and maintaining populations large enough to avoid reclassification as endangered [3]–[6]. Analysis of the relationship between harvest, survival rates and population growth is useful if these objectives (or broader objectives related to predator conservation and ecosystem function) are to be met.

Here, we use previously published data [1], [10] from 21 North American wolf populations (including the recently delisted wolves of the Northern Rocky Mountains) to evaluate relationships between human offtake, mortality and population growth of wolves, and consider the implications for policy.

### Human Offtake and Total Mortality in Wolves

Mortality due to hunting can increase a population's total death rate (additive mortality) or be compensated by density-dependent reductions in non-harvest mortality factors, thus having little effect on overall mortality (compensatory mortality). Williams et al. [11] and Lebreton [12] provide excellent reviews of compensatory and additive mortality. Formally, harvest mortality is fully additive when the regression of total mortality on harvest rate [with slope = 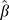 and intercept = 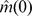] yields 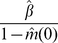 = 1. When 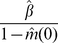 = 0, a harvest is fully compensatory [up to a threshold harvest = 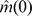, the rate of mortality with no harvest]. A harvest is partially additive when 0<
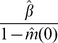
<1, and super-additive when 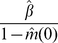
>1. A super-additive harvest increases total mortality beyond the effect of direct killing itself, through social disruption or the loss of dependent offspring.

It is widely argued that human-caused wolf mortality is mainly compensatory, with little effect on wolf dynamics until a large proportion of the population is harvested. Haight et al. [13] summarized that “natural mortality decreases when a wolf population is harvested” and “sustainable harvest rates of 30%–50% have been estimated for free ranging populations” (p. 850). Mech [14] stated that “most human-caused mortality is compensatory” (p.74). In the most comprehensive prior analysis of this question, Fuller et al. [10] concurred that “the principle of compensation operates in wolf populations” (p. 185). Using data from 18 wolf populations, Fuller et al. regressed total mortality on human-caused mortality, and concluded that human-caused mortality was largely compensatory. However, the slope (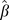 = 0.73) and intercept (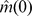 = 0.20) they reported yield 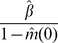 = 0.91, indicating that human harvest was almost fully additive. Thus, there is reason to reconsider the inference that human-caused wolf mortality is primarily compensatory.

## Methods

We tested relationships between the rates of population growth, total mortality and human-caused mortality. To assemble data we began with the 18 populations examined by Fuller et al. [10] in their comprehensive 2003 review. For consistency in the data examined across studies, we used the values that Fuller et al. tabulated (see their Table 6.8) from prior single-population studies, and we retained their decision to divide the data from one population (Isle Royale) into two subsets, based on changes in long-term population trajectory. We tabulated data from United States Fish and Wildlife Service annual reports [1] for wolves in the three segments of the Northern Rocky Mountains (NRM) Recovery Area (Greater Yellowstone, *N* = 11 years, 1998–2008; Central Idaho, *N* = 8 years, 2001–2008, Northwest Montana, *N* = 10 years, 1999–2008). Changes across years in the method of tabulating data in USFWS annual reports yielded different sample sizes for the three segments of the NRM metapopulation. Finally, we used Google Scholar and Scopus to search on the keywords ‘wolf’ and ‘*Canis lupus*’, and for the names of all of the authors of studies tabulated by Fuller et al [10] (their Table 6.8). This search yielded no additional studies with the requisite data. Collectively, these procedures yielded 48 estimates of population growth, harvest rate and total mortality rate from 21 populations (19 estimates as in Fuller et al.'s [10] Table 6.8, and 29 estimates for NRM wolves from USFWS annual reports through 2009 [1]).

**Figure 1:**
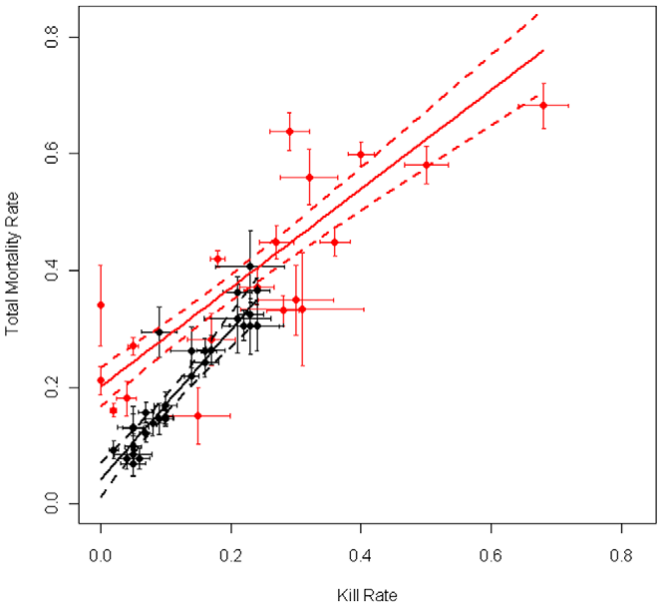
The relationship between total annual mortality and human offtake for wolves in the Northern Rocky Mountains Recovery Area (black) and other populations (red). Points are annual means for the Northern Rocky Mountains data, and multi-year means for other populations. The bars on each point show one standard error. The relationships shown are from the best-supported model in Table 1, a linear relationship with separate slopes and intercepts for the two subsets of data. Dashed lines show 95% confidence bands, accounting for overdispersion by multiplying the variance by the inflation factor (c-hat) from the best-supported model.

**Figure 2:**
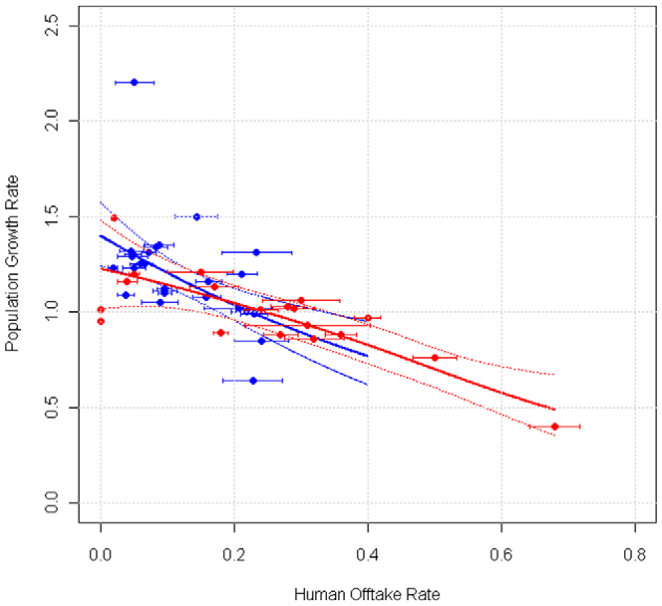
The relationship between population growth (λ) and annual human offtake for wolves in the Northern Rocky Mountains Recovery Area and other populations. Points show annual means for the Northern Rocky Mountains (blue), and multi-year means for other populations (red). Bars show one standard error. Because three models were similarly supported by the data (Table 3), solid lines show the model-averaged function based on all models with Akaike weights ≥0.01. Dashed lines show 95% confidence bands for the model-averaged functions. Blue: Northern Rocky Mountains. Red: Other populations.

Our analyses test two basic hypotheses. First, was total mortality affected by human offtake, and if so, what was the form of the relationship? Second, was the population growth rate (λ) affected by human offtake, and if so, what was the form of the relationship? To test the relationship of harvest to population growth, we evaluated a set of *a priori* models using Akaike's Information Criterion corrected for sample size (AICc). To test the relationship of harvest to mortality (which was approximately binomially distributed), we used quasi-AICc (QAICc) values, with variances adjusted for over-dispersion using the estimated value of c-hat from a quasi-binomial model with a linear link function, and taking the number of population means (*N* = 48) as the sample size to avoid pseudo-replication. Annual reports from the USFWS [1] allowed us to tabulate data from NRM populations as annual means. Data from other populations were multi-year means (following Fuller et al. [10]). We weighted each estimate by sample size to account for variation in the amount of information and the precision of each estimate, and we show the standard error (whiskers) of each population estimate (point) in Figures 1 & 2. Below, we discuss the possible effects of sampling error on the inferences from these models.

Tables 1–3 identify and describe the set of *a priori* models for each analysis. Briefly, each analysis included a set of plausible generalized linear and nonlinear (e.g., breakpoint and general additive models) relationships and tested for regional differences in slopes and intercepts. In each model set, the linear models formalized the hypothesis that human offtake causes additive changes in the rate of survival or population growth, and the breakpoint and general additive models formalized the hypothesis that the effects of offtake are partially or completely compensated. Both model sets included an intercept-only model, to evaluate the explanatory power of the best-supported models in comparison to a null hypothesis of no relationship between harvest and the dependent variable.

**Table 1:** (A) Comparison of models of the relationship between total annual mortality and human-caused mortality for wolves in North America.

Model description^1^	Log Likelihood	K^2^	QAICc^3^	ΔQAICc	ω^4^
i. Regional intercept & slopes	−225.13	5	122.31	0.00	0.69
ii. Gen additive model by region	−212.17	9.02	123.88	1.57	0.31
iii. Breakpoint model by region	−310.69	5	164.99	42.68	0.00
iv. Common intercept & slope	−354.55	3	182.87	60.56	0.00
v. Common breakpoint model	−378.79	3	194.96	72.65	0.00
vi. Single intercept only	−965.62	2	485.70	363.40	0.00

1 Expanded model descriptions:

(i) Generalized linear model (binomial errors with identity link) that allowed different slopes and intercepts for the relationship between total mortality and human offtake for two regions (wolves in the Northern Rocky Mountains (NRM) recovery area and wolves in previously-studied populations),

(ii) General additive model that allowed regional differences, fit in the ‘mgcv’ package of R with cross-validation used to determine the optimum amount of smoothing. GAM models allow curvilinear functions if the data support curvature.

(iii) Generalized linear model (binomial errors with identity link) that allowed the slope to change at a breakpoint and allowed regional differences,

(iv) Generalized linear model (binomial errors with identity link) with no regional effect.

(v) Generalized linear model (binomial errors with identity link) that allowed the slope to change at a breakpoint with no regional effect,

(vi) Constant total mortality (no effect of human offtake on total mortality).

2 Number of parameters in the model (non-integer values are expected for general additive models).

3 *QAICc calculated using c-hat = 4, the estimated overdispersion value obtained from a quasi-binomial model and using the number of mortality rates (*N* = 48) as the sample size.

4 Akaike model weight.

**Table 2 pone-0012918-t002:** Intercepts and regression coefficients from the best model of total mortality as a function of human-caused mortality in North American wolf populations (see Table 1 for model selection using QAICc scores).

Parameter	Estimate	Std. Error	Lower 95% C.L.	Upper 95% C.L.
***Intercept*** 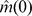				
Northern Rocky Mountains	0.041	0.015	0.011	0.071
Other Populations	0.200	0.017	0.167	0.234
***Slope*** 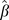				
Northern Rocky Mountains	1.285	0.127	1.036	1.534
Other Populations	0.849	0.069	0.714	0.983

This is a generalized linear model (binomial errors, identity link) with a linear relationship between total mortality and human-caused mortality, and regional differences in the parameters of this relationship.

**Table 3:** Comparison of models of the relationship between annual population growth and human-caused mortality for wolves in North America.

Model description^1^	Log Likelihood	K^2^	R^2^adj^3^	ΔAICc	ω^4^
i. General additive model by region	20.63	6.15	0.59	0.00	0.63
ii. Common intercept & slope	15.92	3	0.53	1.40	0.31
iii. Regional intercept & slopes	16.64	5	0.52	4.91	0.05
iv. Regional intercepts, no slopes	2.29	3	0.14	28.66	0.00
v. Single intercept only	−1.51	2	0.00	33.96	0.00

1 Expanded model descriptions:

(i) General additive model (GAM) that allowed regional differences, fit in the ‘mgcv’ package of R with cross-validation used to determine the optimum amount of smoothing. GAM models allow curvilinear functions if the data support curvature.

(ii) General linear model (normal errors with log link) with no regional effect on slope and intercept.

(iii) General linear model (normal errors with log link) that allowed regional differences in the slope and intercept.

(iv) Constant total mortality (no effect of human offtake on total mortality), with regional differences.

(v) Constant total mortality (no effect of human offtake on total mortality).

2 Number of parameters in the model (non-integer values are expected for general additive models).

3 The coefficient of determination (R^2^) adjusted for degrees of freedom.

4 Akaike model weight.

From the perspective of collating data for meta-analysis, we did not suspect that reporting bias against ‘negative’ results would be an important issue for the publication of data on rates of harvest, total mortality or population growth, because most of the original studies were descriptive in nature, and for the Northern Rockies, raw data were reported in a standardized fashion in annual reports. For most of the original studies, it is likely that some wolves were killed illegally and not reported. Because illegal killing cannot be quantified, our analyses are based on reported offtake (which is a rational basis for management decisions about wolf harvest quotas). For data from NRM populations [1], we included ‘missing’ radiocollared animals (but not known dispersers) in the number of total deaths. With this method, any undetected illegal killing of a radiocollared wolf would contribute to the estimated total mortality rate but not to the estimated human killing rate. However, the number of missing wolves was a small proportion (typically 5–10%) of known mortality, and large carnivores go missing for reasons other than illegal killing (e.g., failure of VHF transmitters, long distance dispersal, natural mortality with transmitter damage). For non-NRM populations, methods of monitoring varied, so the extent and direction of biases due to unreported illegal killing is unknown. Issues related to unreported harvesting and the dynamics of wolves merit further study.

## Results and Discussion

### Human Offtake and the Annual Mortality Rate

There is a strong association between human offtake and total mortality rate across North American wolf populations. The best-supported model of the relationship between total mortality and human caused mortality was linear, with slopes that differed for wolves in the NRM and elsewhere (Table 1). Human-caused mortality has been lower for NRM wolves than in most other populations (Fig. 1) but has exceeded 20% killed in some years through predator control, while under Endangered Species Act regulations. From the best model (Fig. 1), 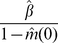 was 1.34 for NRM wolves (96% CI: 1.11 to 1.56, after inflating variances to account for estimated overdispersion) and 1.06 (95% CI: 0.92 to 1.20, again adjusted for overdispersion) for other populations (Table 2). These results suggest that mortality due to humans was not compensatory but highly additive or even super-additive. Super-additivity might be expected from the consequences of breeder mortality in wolves [15]. In a study of 10 populations, pup survival declined with decreasing pack size, 38% of packs disbanded following loss of a breeder, and only 47% of packs that lost a breeder reproduced in the subsequent year (9% reproduced after loss of both breeders) [15]. These consequences of social disruption are sufficiently large to compound the direct effect of mortality due to hunting, particularly when packs are small, so that a high proportion of adults are breeders. In 2008, 120 (69%) of 173 packs in the NRM held 4 or fewer adults [1], so that randomly killed adults would have ≥50% probability of being breeders. If these mechanisms do underlie super-additivity, the full effects of harvesting might not be manifest until the following year (or longer).

Models of compensatory mortality predict that the total mortality rate is initially constant as harvest increases, and then begins to rise above a threshold harvest rate equal to 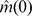. Contrary to this prediction, models with a change in slope (breakpoint and general additive models) did not fit the data well as linear models (Table 1). A general additive model fit only slightly worse than the linear model (Table 1), but its curvature was slight, and in the direction opposite that predicted by a model of compensatory mortality. These results provide further evidence that human-caused mortality was additive rather than compensatory. Finally, harvest can only be compensatory (in the sense of ‘competing risks’) when the rate of offtake is less than or equal to the rate of mortality in the absence of harvest, 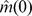, but mortality rates in the absence of harvest are low for wolves (as for most long-lived large mammals). Using estimates from the best model (Table 2), 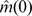 was 0.04±0.015 (SE) for the NRM and 0.20±0.017 for other populations, so there was little scope for harvest mortality to be compensatory, especially for NRM wolves.

A recent re-analysis of the data for non-NRM populations [16] also concluded that ‘human take does not share a compensatory interaction with natural mortality’, because natural mortality did not decline with increasing human offtake. A recent analysis of the correlates of mortality in a large sample of radiocollared NRM wolves [17] reported that human killing accounted for a minimum of 54% of wolf mortality between 1982 and 2004, but did not directly test the relationship between human offtake and total mortality.

In studies that examine responses to harvest at a relatively small spatial scale, immigration can compensate for mortality due to harvest [10], [16]. However, this mechanism is fundamentally different than compensatory reductions in non-harvest mortality, because compensatory immigration simply involves movement of individuals onto a study site from locations off of the study site. When we consider the dynamics of the entire population, this movement does not truly compensate for harvest mortality, because gains in one pack are offset by losses in another. Indeed, if dispersing wolves have lower rates of survival than pack-living wolves (as in other social carnivores [18]), then an increase in dispersal would further reduce mean survival for the population as a whole, rather than compensating.

### Human Offtake and Wolf Population Growth Rates

Given that mortality due to hunting was strongly additive or super-additive, we tested the effect of harvest on population growth rates, an analysis that incorporates the possibility that reproduction might increase to offset human-caused mortality. The literature on wolf harvesting includes many estimates of the proportion of a wolf population that must be killed to reduce wolf numbers. These studies often conclude that a harvest of 28%–50% of a wolf population is required to make a population decline. For example, Mech (2001) stated that “wolf populations can sustain annual winter harvest rates of 28%–47%” (p. 74), and “it is important for all to recognize that a moderate to large kill of wolves from the general population will have little limiting or reducing effect on the population” (p. 75) [14]. Adams et al. [16] concluded that “population trends were not correlated with annual human take ≤29%” (p. 1). With respect to policy, the 2003 delisting decision by the USFWS [2] stated that “the levels of documented human-caused mortality in the Northern Rocky Mountains have not, at this time, been significant enough to cause declines in the wolf population *or to slow overall wolf population growth*” ( p. 15851, emphasis added). Mirroring these conclusions, state management plans for NRM wolf populations [3], [5] state that “wolf populations can apparently withstand human-caused mortality of 28%–50% without declining” (Idaho) and “wolf populations can apparently withstand human-caused mortality rates of 28%–35% without declining” (Montana). Why the state policies identify different upper limits is not clear, but the policies concur that harvests up to 35% are sustainable. The federal policy goes further, stating that human offtake has not slowed population growth in NRM wolves.

**Figure 3:**
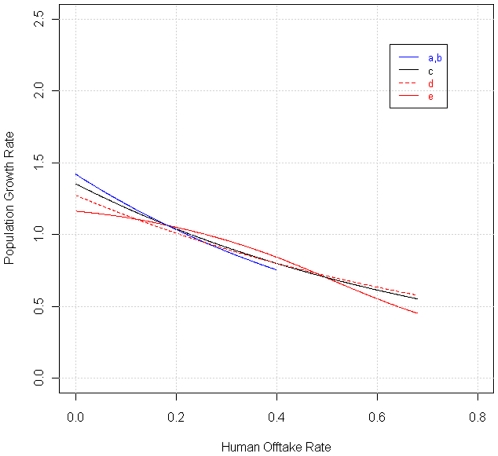
The individual models that were averaged to produce the functions in **Figure 2** were highly congruent in their estimates of the offtake that yields λ = 1. a,b: GLM and GAM for Northern Rockies (these models were identical), c: GLM for all data combined, d,e: GLM and GAM for other populations.

We evaluated these statements using information theory to compare models of population growth (λ) as a function of human harvest, for NRM wolves and other populations (Table 3). All models supported by the data (Table 3) showed that population growth declined across all observed levels of human-caused mortality, which included low levels (Fig. 2). Because three models had reasonable support from the data (Table 3), we used model averaging (Figs. 2 & 3) to estimate the maximum offtake expected to yield λ≥1. For NRM wolves, the maximum stable offtake was 0.224 (model-averaged 95% CI: 0.177–0.335). For other populations, the maximum stable offtake was 0.245 (model-averaged 95% CI: 0.149–0.343). These estimates coincide well with the simple observation that NRM wolf populations have declined three times in the past decade, in each case with human harvests of 23%–24% (Fig 2). Better understanding of harvest effects can help managers achieve population goals. In July 2010, the Montana Fish Wildlife and Parks Commission approved an increase in the wolf harvest from 75 to 186 wolves. On the basis of internal analysis, the Montana Department of Fish Wildlife & Parks predicted that this harvest would, in combination with predator control killing continuing at past levels, cause a 13% decrease in wolf numbers. A harvest of 186 wolves together with 145 killed through predator control would yield a total offtake of 331 wolves, or 63% of the Montana population (which was estimated to number 524 at the end of 2009). The data in Fig 2 suggest that a direct killing rate of 0.63 would typically produce a decline substantially greater than 13%.

Because wolf populations in the Northern Rocky Mountains have grown since reintroduction, we tested whether growth slowed as population sizes increased. Overall, the NRM population has increased 15-fold over the past 15 years, providing unusually broad scope to test for density-dependent changes in the growth rate. Despite this, population growth was not detectably related to population size in the Northern Rocky Mountains (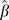 = −0.06±0.15 S.E., Wald statistic = 0.19, P = 0.66), and a model of linear density dependence was 5.5 AICc units worse than a model of linear harvesting effects on population growth. Density-dependence underlies compensation, so these observations reinforce the expectation that harvesting is not likely to increase reproduction or decrease natural mortality by reducing competition for resources, within the range of wolf densities seen to date. Although the data to date do not reveal clear density dependence, simply inspecting the growth curve gives some indication that NRM population growth may have slowed since 2007 [1]. If so, a reduced growth rate might indicate the incipient emergence of density dependent growth driven by resource competition. Contrary to this hypothesis, the survival of radiocollared NRM wolves increased with population density [17], rather than decreasing as would be expected with density dependent growth. Slower growth since 2007 could also be due to increased offtake by humans, if the rate of offtake is positively related to population density (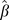 = 0.08±0.05 S.E., Wald statistic = 2.69, P = 0.10). Between 1982 and 2004, human killing accounted for a minimum of 54% of total mortality for radiocollared NRM wolves [17], revealing that human offtake was more strongly limiting than all other factors combined, at least with respect to survival. (Anthropogenic effects are a dominant limiting factor for many large carnivores, world-wide.) If human offtake holds wolves at densities below the region's ecological carrying capacity, then it is plausible that density dependence will remain weak or equivocal.

Our analysis is based on comparison of multiple populations, rather than changes through time in a single population. Prior studies of human harvesting and its effect on wolf dynamics [10], [16] were also based on comparison across populations, so the differences in our inferences are not due to this distinction. Comparisons across populations have a broader scope of inference than single-population studies, but correlations across populations can be affected by uncontrolled heterogeneity among sites. By including models that allowed different slopes and intercepts for NRM wolves and other populations, we incorporated heterogeneity to the degree possible with the data in hand. We encourage further research to test whether human offtake still appears to be largely additive or super-additive with hierarchical models that more completely account for differences among populations.

Our results confirm that wolf populations can grow while being harvested. However, point estimates for the maximum offtake rate associated with stable wolf populations are below the thresholds identified by recent state wolf management plans. Moreover, sustainable harvest is probably lower than our estimates, for two reasons. First, our models are based on deterministic estimates of population growth, which typically over-estimate true stochastic growth rates [19]. Second, estimated human offtake has an associated variance in these data (Fig. 2), and the effect of variance in an independent variable is to bias a regression's slope toward zero. For these reasons, we encourage further work on this topic, especially analysis with direct data on the survival of known individuals.

The management of wolves is controversial, and recent experience in the Rocky Mountains shows that any policy will face opposition from at least one constituency. Different stakeholders desire different numbers of wolves on the landscape. In structured decision-making it is important to isolate ecological analysis that considers the likely outcome of a policy from the discussion that considers whether or not that outcome is desirable [20]. Here, we have attempted to correct several broad misconceptions about the quantitative relationships between harvest intensity, mortality and population growth rates of wolves. The meta-analysis suggests that the effect of human-caused mortality on wolf dynamics is greater than suggested by current management plans (see references [21], [22] for similar recent inferences about the role of human offtake in the dynamics of large felids including African lions, *Panthera leo*, and North American cougars, *Panthera concolor*). These results should help to inform wolf management, in conjunction with other important considerations about the interactions of wolves with ungulate prey, livestock, people, and ecosystems.

### Conclusions

In summary, it appears that: (1) Wolves can be harvested sustainably within limits. (2) Examined across populations, human killing of wolves is generally not compensatory, as has been widely argued. Management policies should not assume that an increase in human-caused mortality will be offset by a decline in natural mortality. (3) Rather, the effect of harvesting on wolf mortality appears highly additive to super-additive. Evidence for super-additive mortality is stronger for wolves in the recently-delisted Northern Rocky Mountains Recover Area, which often live in small packs. (4) Estimated sustainable harvest levels from this meta-analysis are lower than current Northern Rocky Mountain management plans suggest, and lower than the 2009 rate of offtake for the Northern Rockies. While some wolf populations might maintain constant population size at the harvest intensities considered sustainable by current state management plans, our results suggest that such harvests will generally cause wolf populations to decline. (5) The relationship of population growth rates to killing rates suggest that a proposed 2.5-fold increase in wolf harvest for 2010 is likely to reduce population size by a greater amount than management policy statements for Montana have stated. (6) The effects of harvesting on population growth may not be fully manifest in one year. These results should help with the development of policies for the management of wolves, particularly newly-delisted wolf populations in the Northern Rocky Mountains. The basic point that harvest mortality cannot be highly compensatory via substitution of mortality under conditions of low natural mortality (as in most long-lived species [12]) should be clearly expressed in policies for the management of large carnivores. Finally, these results highlight the ongoing need to fully incorporate quantitative analysis of available data in the development of conservation and management policies.
